# What Should Be the Relation of Our Government to the Dental Profession?

**Published:** 1902-03

**Authors:** Charles F. Allan

**Affiliations:** Newburgh, N. Y.


### International Dental Journal.

Vol. XXIII. March, 1902. No. 3.

### Original Communications.[1]

### WHAT SHOULD BE THE RELATION OF OUR GOVERNMENT TO THE DENTAL PROFESSION?[2]

BY DR. CHARLES F. ALLAN, NEWBURGH, N. Y.

It is perhaps not too broad a statement to say that the healing art stands nearer to the individual, is of more importance to his well-being, and consequently to the well-being of the State, than any or all of the learned professions.

The head of the nation, the governor of the State, the legislator in Congress, the judge on the bench, each is dependent at times on the doctor's good services, and whether he is to die or in the future be a tower of strength to the nation or the community he serves depends solely on the ability and earnestness of the physician in charge.

This dependence of the individual, and consequently of the State, which is but the aggregation of its individuals, on the members of the healing art is recognized more or less officially by all civilized governments in Europe; and exceptional ability and great discov-

[1] The editor and publishers are not responsible for the views of authors of papers published in this department, nor for any claim to novelty, or otherwise, that may be made by them. No papers will be received for this department that have appeared in any other journal published in the country.

[2] Read before The New York Institute of Stomatology, November 8, 1901.

eries, or new methods of practice promising great returns, are rewarded in these governments by titles or memberships in orders which have very practical significance and value to the medical men so honored.

The great physician is always considered as honoring and ennobling his country, and the heads of the State are always ready to do him honor.

Moreover, there are international features connected with the support foreign governments give to men of science, and especially to those medically educated; and it is always the case at the meetings of the international medical congresses in Europe, that more or less the gathered physicians from other lands are made the guests of the nation visited, and are officially honored.

At the meeting of the International Congress in Russia, but a couple of years or so ago, the delegates were welcomed officially to the land of the Czar; social and peculiar privileges were showered upon them; the facilities of the state railroads were in some instances put at their disposal free of any expense, and in many ways the visitors were welcomed as the guests of the nation.

At the meeting in Rome, several years ago, the King of Italy and his cabinet took great pains to emphasize their official welcome, and many social functions were given in their honor. It was the same in Berlin a few years ago, and in Paris last year, the visiting physicians receiving special honors and being welcomed largely as the guests of the nation. These nations go on record as if to emphasize, in the beautiful words of Samuel Smiles, "The career of a great man remains a monument of human energy, his thoughts and acts survive and leave an indelible stamp upon his race. Thus the spirit of life is prolonged and perpetuated, moulding the thought and will and thereby contributing to form the character of future generations;" and thus it is that this "great man," so honored, honors the country that honors him.

This is the spirit of the civilized countries of the world, with the exception, sad to say, of our own government, which, instead of giving honor and a recognition of ability, alone of all the great nations, taxes medicines, surgical and dental instruments and supplies coming from abroad, and, as much as the government can, makes a Chinese wall against the adoption and use of methods, instruments, and materials proved of value in other countries.

The United States has the unenviable distinction of having a tariff duty on dental materials and instruments higher than that of any other country, though our manufacturers have less to fear from competition than any in the world.

This, as has been well said, "has been brought about by the manufacturers acting as doctors for the needs and interests of the dental profession." It must be conceded that free trade in dental and surgical instruments is certainly in the best interest of science, and works for the benefit of humanity; yet the United States leads the world with a duty of forty-five per cent. on dental instruments, while Great Britain, France, Germany, Belgium, Canada, and Mexico have no duties, and in no other country is a higher duty than ten per cent. imposed. In fact, the tariffs of all the different countries of the world added together are but a trifle, if any, more than that imposed by the United States alone. I emphasize this because I feel that it is not generally understood how alone the United States stands in the imposition of this onerous tax on science and the healing art.

The markets of the world are practically free to the manufacturers in this country of dental goods, and all over the world our manufacturers compete, seemingly on even terms, with the cheap, or perhaps I should say "pauper labor" of European countries; and presumably making their greatest profits on the higher rates charged American dentists. This too, in the face of the fact that our government is now facing a surplus of seventy million dollars and more per year, and has no need of the revenue. Surely, it is high time that our government ceased this oppression.

From the nature of our institutions it is probably impracticable for our government to reward great professional merit and large scientific attainments in the way they are rewarded in the countries of the Old World; but it is not impracticable to take off oppressive taxes and show, at least negatively, that the government is not in opposition to professional success and scientific progress.

We do not ask favor, we only want hands off by the government so that we can buy medicines, materials, and instruments in the markets of the world where we can get them cheapest and best, so that we can take advantage immediately of pharmaceutical, scientific, and mechanical aids to our profession.

As a nation we have been advancing in manufactures with giant strides the past few years; pure science, scientific researches made for scientific purposes alone have been applied by our manufacturers in most practical ways, making vast economies, so that in many lines we now lead the world; but possibly in the domain of applied chemistry we still have to acknowledge the lead of German chemists.

In the manufacture of aniline dyes Germany in the last decade has practically wrested the whole business from England, business amounting to many millions of dollars, and precedence still has to be given to the German manufacturing pharmacists; but every ounce of chemicals entering our country has to pay tribute to our paternal government, and that intimate relation that should exist between pharmacal manufacturers and those who use their products is prevented by the Chinese wall our government places around the medical profession and its best interests. In the line of dental and surgical instruments the prohibition of those of foreign make is almost absolute.

Nearly a year ago I wrote to the New York house of Ash & Sons for some steel- and diamond-pointed burs, made with especial reference to inlay work; also I wanted to see and examine other of their wares advertised in the journal published by their house; but I got a reply from them saying that they did not carry the goods in stock, and would have to order from their London house. I wrote a letter deprecating this lack of enterprise, and I also wrote the parent house in London. The instruments were special instruments, made only, so far as I know, by the firm mentioned. From both houses I got very courteous replies, saying that the high duty of forty-five per cent. practically prevented their importing and keeping in stock the line of goods they wanted to. Their goods cost them practically as much to manufacture in England as they would in this country, and the forty-five per cent. duty meant prohibition.

American dentists are very practical; they know about how much instruments and materials ought to cost, but they practically know nothing of the workings of the prohibitive tariff.

European dentists, perhaps I should say American dentists in Europe, are far ahead of American dentists in America in the use of porcelain as a filling-material; it has, to a large extent, been forced on them by the great dislike of their patients to the show and glitter of gold, and now that *we* see the immense æsthetic and practical advantage the material has, we would be glad to learn from them and take advantage of every mechanical aid they have found useful. But *no;* our government says *no,* unless we pay tribute to our already rich and prosperous dental manufacturers.

Possibly in no line of manufacture in our country have business acumen and mechanical ingenuity and ability been wedded together in the accomplishment of better results than in the making of dental and surgical instruments, appliances, and materials; in no line of work do manufacturers need protection less from foreign manufacturers; and, as a matter of fact, our makers send their goods to all parts of the world in open competition in price with those of foreign make. If our manufacturers do not need protection in foreign countries against the foreign maker with his so-called "pauper labor," why should he need protection in his own market? Why can he not sell as cheaply to us as he does to them?

We are proud of the beautiful goods our manufacturers turn out, and they are so good that they need no protection; we would buy but little less from them if the tariff were removed, but that "little less," those new and occasionally very good ideas that have so far found no counterpart in our country, we ought to be able to get as soon as they appear in the market, and we ought to be able to get them, as men engaged in the healing of the nation, without paying unnecessary tax to the already over-liberal revenues of our government.

The tariff on dental and surgical goods is not protective in any sense. We ought not to have to pay any tariff even if it were protective, but, as a matter of fact, it is only an unnecessary act of prohibition against the whole of the healing art, and such tariff should have no place in our legislative enactment.

I am told that Polk's lists of dentists are the most reliable of any we have, and according to him there are about twenty-five thousand dental practitioners in this country, and, of course, there are still more thousands of physicians in general and special practice (at least a hundred and fifty thousand).

Now, in our own specialty does it not seem hard that twenty-five thousand dentists should be hampered in their practice, should be prohibited from free access to the markets of the world, solely to protect (so they call it) the infant industry of a few very small firms and one large corporation? (The latter the first in the world established on a large scale.) Have medical and dental practitioners any rights under the government in this matter? It would seem not.

In my prefatory words I spoke of the high honor paid the medical profession in all its branches by the governments of other countries, and it has been an unpleasant duty to point out as well that our government has had for us only oppressive taxes. I can but feel that this tariff question, in so far as it affects scientific men, especially the medical profession, is but little understood, and that it needs only that light shall be thrown on it and properly brought before the country for remedial legislation to follow.

It is in this connection that I present to you and the profession the facts embodied in the sheets that have been distributed among you. These statistics of the tariff duties of most of the countries of the world represent an immense deal of labor, and are thought to be entirely reliable. Some countries have a specific duty in dental goods, other countries have an *ad valorem* duty, and still others both specific and *ad valorem.* But for the sake of comparison, and that the matter may be entirely lucid, the rates have all been put on an *ad valorem* basis and reduced to our own currency.

As the popular phrase goes, it is now *"up to you" for action.*

During most of the career of our late martyred President the "Protection of American Industries" was a phrase which to him represented almost a fetish to be worshipped, yet, in the fulness of the years of his executive duties and with the responsibility of the well-being of all the people of this country pressing on him, he uttered these words of extreme wisdom and great statesmanship, in his Buffalo address, that endeared him in a peculiar way and pre-eminently with all the people of our land.

"We must not repose in tranquil security that we can forever sell everything and buy little or nothing. . . . Reciprocity is the natural outgrowth of our wonderful industrial development. . . . The period of exclusiveness is past, the expansion of our trade and commerce is the pressing problem. . . . Reciprocity treaties are in harmony with the spirit of the times; measures of retaliation are not."

And then! With these and other words of wisdom and love for all his people fresh from his lips, the assassin kills him. Surely he who runs may read the lesson of these good words and that untimely end.

A former ambassador of the court of St. James has eloquently said, "I believe the only doctrine for a man or a nation is to do a friend the utmost benefit and an opponent no more harm than

STATISTICS OF TARIFFS ON DENTAL GOODS IN VARIOUS COUNTRIES, COMPILED ON AN AD VALOREM BASIS.

| | United States. | Great Britain. | Canada. | Germany. | France. | Italy. | Austria and Hungary. | Russia. | Denmark. | Norway. | Sweden. | Spain. | Switzerland. | Belgium. | Holland. | Romania. | Turkey. | Greece. | Mexico. |
|---|---|---|---|---|---|---|---|---|---|---|---|---|---|---|---|---|---|---|---|
| Porcelain teeth | 60 | No duties on dental goods. | 20 | 1 | 1 | 1 | 1 | 1 | 1 | 1 | 15 | 1 | 1 | 15 | 5 | 1 | 8 | No duties, but a small octroi is charged into Athens. | 2½ |
| Mineral teeth | 35 | | 20 | 1 | 1 | 1 | 1 | 1 | 1 | 1 | 15 | 1 | 1 | 15 | 5 | 1 | 8 | | 2 |
| Dental rubber | 30 | | 25 | 1 | 5 | 2½ | 2½ | 15 | 2 | Free | 8 | 5 | 2½ | 10 | 5 | 2½ | 8 | | 15 |
| Dental cement | 21 | | 20 | 1 | 5 | 1 | 1 | 5 | 1 | Free | 15 | .... | 2½ | Free | 5 | 3 | 8 | | 5 |
| Dental amalgam | 45 | | 30 | 2 | 5 | 1 | 1 | 1 | 1 | Free | 15 | .... | 1 | Free | 5 | 1 | 8 | | 2 |
| Gutta-percha (filling) | 35 | | 25 | 1 | 1 | 1 | 1 | 11 | 1 | Free | 1 | 1 | 1 | 10 | 5 | Free | 8 | | 2 |
| Gold-foils and cylinders | 45 | | 30 | 1 | 1 | 1 | 1 | 6 | 1 | Free | 1 | 1 | .... | 5 | 5 | 1 | 8 | | 1 |
| Dental instruments | 45 | | Free | Free | Free | 10 | 2½ | 2½ | 5 | 2½ | 10 | 2½ | 1 | Free | 5 | 1 | 8 | | Free |
| Dental engines | 45 | | Free | 2½ | 2½ | 10 | 7 | 2½ | 1 | 2½ | 10 | .... | .... | 5 | 5 | 2½ | 8 | | .... |
| Dental chairs | 45 | | 30 | 7½ | 2½ | 5 | 8 | 5 | 8 | 5 | 10 | .... | .... | 5 | 5 | 10 | 8 | | .... |
| Dental cabinets | 45 | | 30 | 7½ | 2½ | 10 | 5 | 20 | 8 | 10 | 20 | .... | .... | 5 | 5 | .... | 8 | | .... |
| Dental tools | 45 | | 30 | 3 | 2½ | 10 | 2½ | 5 | 2½ | Free | 10 | .... | .... | 5 | 5 | 2 | 8 | | .... |
| Vulcanizers | 45 | | 30 | 7½ | 10 | 7½ | 5 | 10 | 2½ | 3 | 10 | .... | .... | 5 | 5 | 25 | 8 | | .... |
| Furnaces | 45 | | 30 | 5 | 2 | 5 | 5 | 5 | 2 | 2½ | 10 | .... | .... | 5 | 5 | 15 | 8 | | .... |
| Gas apparatus | 45 | | 30 | 5 | 2½ | 2½ | 5 | 7½ | 8 | 2½ | 10 | .... | .... | 5 | 5 | 10 | 8 | | .... |
| Modelling compound wax preparation | 25 | | 20 | 2 | 10 | 7½ | 2½ | 5 | 5 | Free | 3 | .... | 7½ | 10 | 5 | 5 | 8 | | .... |

necessary. I cannot believe in the advancement of any country founded on the misfortune or injury of another."

Do you notice how beautifully these words fit in with the golden rule of reciprocity as it is found in the Buffalo address of President McKinley? And do you not see how superlatively appropriate it would be for our legislators now to allow the representatives of the healing art in this country to import, freely and without duty, everything from foreign countries necessary to their progress and their best interests, thereby reciprocating that which practically every nation of the world allows to us?

